# Knowledge-rich temporal relation identification and classification in clinical notes

**DOI:** 10.1093/database/bau109

**Published:** 2014-11-19

**Authors:** Jennifer D’Souza, Vincent Ng

**Affiliations:** Human Language Technology Research Institute, University of Texas at Dallas, Richardson, TX 75083-0688

## Abstract

**Motivation:** We examine the task of temporal relation classification for the clinical domain. Our approach to this task departs from existing ones in that it is (i) ‘knowledge-rich’, employing sophisticated knowledge derived from discourse relations as well as both domain-independent and domain-dependent semantic relations, and (ii) ‘hybrid’, combining the strengths of rule-based and learning-based approaches. Evaluation results on the i2b2 Clinical Temporal Relations Challenge corpus show that our approach yields a 17–24% and 8–14% relative reduction in error over a state-of-the-art learning-based baseline system when gold-standard and automatically identified temporal relations are used, respectively.

**Database URL:** http://www.hlt.utdallas.edu/~jld082000/temporal-relations/

## Introduction

Temporal relation classification, one of the most important temporal information extraction (IE) tasks, involves classifying a given event–event pair or event–time pair in a text as one of a set of predefined temporal relations. The creation of the TimeBank corpus (1), as well as the organization of the TempEval-1 (2) and TempEval-2 (3) evaluation exercises, has facilitated the evaluation of temporal relation classification systems for the news domain.

Our goal in this article is to advance the state of the art in temporal relation classification. Although virtually all previous work on this task has focused on the news domain, we work with a relatively unexplored domain, the ‘clinical domain’, using the i2b2 Clinical Temporal Relations Challenge corpus (henceforth the *i2b2 corpus*).^1^ To date, this corpus is only accessible to and has only been experimented on by the participants of the Challenge (henceforth the *shared task*).

Our work differs from existing work with respect to both the ‘complexity’ of the task we are addressing and the ‘approach’ we adopt. Regarding task complexity, rather than focus on ‘three’ temporal relations as in the shared task (see the Corpus section for more information), we address an arguably more challenging version of the task where we consider all the 12 relations originally defined in the i2b2 corpus.

Our approach to temporal relation classification can be distinguished from existing approaches, including those developed for the news domain and the clinical domain, in that it involves a large-scale expansion of the linguistic features made available to the classification system. Existing approaches have relied primarily on morpho-syntactic features, as well as a few semantic features extracted from WordNet synsets and VerbOcean’s (4) semantic relations. On the other hand, we propose not only novel lexical and grammatical features, but also sophisticated features involving semantics and discourse. Most notably, we propose (i) discourse features encoding Penn Discourse TreeBank (PDTB) style (5) discourse relations and (ii) semantic features encoding a variety of semantic relations extracted from general-purpose lexical databases such as Propbank and the Merriam-Webster dictionary and (iii) semantic features encoding different types of semantic relations specifically designed for the medical domain.

We employ a system architecture in which we combine a learning-based approach and a rule-based approach. Our motivation behind adopting a hybrid approach stems from our hypothesis that better decision rules can be formed by leveraging human insights to combine the available linguistic features than by using fully automatic machine learning methods.

We evaluate our knowledge-rich, hybrid approach to temporal relation classification in two settings. In the first setting, we assume that we are given event–event and event–time pairs that are known to belong to one of the 12 predefined temporal relations in the i2b2 corpus, and hence the task is to label each pair with one of these 12 relation types. To make things more challenging, however, we assume in the second setting that we are given event–event and event–time pairs that ‘may’ or ‘may not’ belong to one of the 12 relation types. Hence, the task in this setting involves both ‘identifying’ and ‘classifying’ temporal relations. For this task, we first employ a ‘relation identification’ system to determine whether a pair has a relation, and then use the same ‘relation classification’ system as the one in the first setting to classify all and only those pairs that are determined to have a relation by the identification system. Conducting experiments with both settings can enable us to determine how much performance deterioration can be attributed to ‘identifying’ rather than ‘classifying’ temporal relations.

Experiments on the i2b2 corpus show the effectiveness of our approach: under the first and the second settings, it yields a 17–24% and 8–14% relative error reduction, respectively, over a state-of-the-art learning-based baseline system.

Further, we map the 12-class classification results of our system back to their broader 3-class counterparts, and in-turn verify that our system improves on the state-of-the-art 3-class classification results reported in the 2012 i2b2 challenge.

To our knowledge, we are the first to (i) report results for the 12-class temporal relation classification task on the i2b2 corpus; (ii) successfully employ automatically computed predicate–argument relations, medical semantic relations and PDTB-style discourse relations to improve performance on this task and (iii) show that a hybrid approach to this task can yield better results than either a rule-based or learning-based approach. In addition, we release the complete set of rules that we mined from the i2b2 corpus and used in our rule-based approach,^2^ hoping that our insights into how features can be combined as decision rules can benefit researchers interested in this task.

## Corpus

For evaluation, we use the i2b2 corpus, which consists of 310 de-identified discharge summaries pre-partitioned into a training set (190 summaries) and a test set (120 summaries). Each summary is composed of two sections. The first section was created when the patient was admitted and reports History of Present Illness (i.e. her clinical history). The second section was created when the patient was discharged from the hospital and reports Hospital Course. In each summary, the ‘events’, ‘times’ and their ‘temporal relations’ are marked up. An event can be a verb phrase (VP), an adjective phrase, a noun phrase or sometimes an adverb that semantically refers to clinically relevant patient-related actions, contains various attributes, including the ‘type’ of event (see Table 1 for a listing of the six types of events), ‘polarity’ and ‘modality’. A time expression has a type attribute, which specifies whether it is a date, time, duration or frequency, and its value is normalized based on TIMEX3. A temporal relation can be an ‘anchor’ relation, which anchors an event to a time expression (as in Sentence (1)), or an ‘order’ relation, which orders two events (as in Sentence (2)).

1. He was ready for *discharge* home on *postoperative day 3*.
2. She has not *complained* of any *fever*.

**Table 1.** List of event types in the i2b2 corpus

Event type	Example
Test	CT scan
Problem	the tumor
Treatment	Operation
Clinical departments	ICU
Evidential information	Complained
Clinically relevant occurrence	Discharge

Each temporal relation has a type. For example, the relation defined on ‘discharge’ and ‘postoperative day 3’ in (1) has type **Simultaneous**, whereas the relation defined on ‘complained’ and ‘fever’ in (2) has type **Overlap_After**. A temporal relation is defined on an ‘ordered’ pair: in (2), the pair (complained, fever) has type **Overlap_After**, whereas the pair (fever, complained) has type **Before_Overlap**.

Twelve relation types are defined and used to annotate the temporal relations in the i2b2 corpus. Table 2 provides a brief description of these relation types and the relevant statistics.

**Table 2.** The 12 temporal relation types in the i2b2 corpus

Id	Relation	Description	Total (%)	E–E	E–T
1	Simultaneous	*e*_1_ and *e*_2_ happen at the same time or are temporally indistinguishable	4589 (32.5)	3551	1038
2	Overlap	*e*_1_ and *e*_2_ have overlaps in their occurrences but do not happen at the same time	5681 (40.2)	4713	968
3	Before	*e*_1_ happens before *e*_2_ in time	1572 (11.1)	1439	133
4	After	*e*_1_ happens after *e*_2_ in time	577 (4.1)	497	80
5	Before_Overlap	*e*_1_ happens prior to and continues at the time of *e*_2_	506 (3.6)	473	33
6	Overlap_After	*e*_1_ overlaps with and happens after *e*_2_ begins	1642 (11.6)	1584	58
7	During	*e*_1_ persists throughout duration *e*_2_	386 (2.7)	220	166
8	During_Inv	*e*_2_ persists throughout duration *e*_1_	640 (4.5)	522	118
9	Begins	*e*_1_ marks the beginning of *e*_2_	782 (5.5)	591	191
10	Begun_By	*e*_2_ marks the beginning of *e*_1_	204 (1.4)	65	139
11	Ends	*e*_1_ marks the end of *e*_2_	318 (2.3)	197	121
12	Ended_By	*e*_2_ marks the end of *e*_1_	434 (3.1)	293	141

Each relation type is defined on an ordered pair (*e*_1_,*e*_2_), where *e*_1_ and *e*_2_ can each be an event or a time. The ‘Total’ and ‘%’ columns show the number and percentage of instances annotated with the corresponding relation type in the corpus, respectively, and the ‘E–E’ and ‘E–T’ columns show the breakdown by the number of event–event pairs and event–time pairs.

As mentioned in the Introduction section, our approach will be evaluated in two settings: in the first setting, we employ gold-standard temporal relations, and in the second one, we employ automatically identified temporal relations. In both settings, we follow the i2b2 temporal Challenge TLINK track and assume that gold events and time expressions are given.

Unlike the shared task, which focuses on three broad relation types (Overlap′, Before′, After′), our system development focuses on the 12 relation types originally used to annotate the i2b2 corpus. Note that the three broad relation types are created by merging ‘similar’ relation types as follows: (i) **Overlap**′ is composed of **Overlap**, **Simultaneous** and **During**; (ii) **Before**′ is composed of **Before**, **Before_Overlap** and **Ended_By** and (iii) **After**' is composed of **After** and **Begun_By**. Each instance from the remaining four relations is merged into one of the three broad relation types by inverting the order of its elements. For example, if a relation instance (*e*_1_, *e*_2_) is annotated as **Ends**, it is first replaced with the instance (*e*_2_, *e*_1_) with class **Ended_By** and then re-labeled as **Before**′. Thus, classifiers developed for the shared task are only presented with test instances belonging to one of the three broad relation types. On the other hand, our 12-class task is arguably more challenging, since our system has to distinguish not only a relation type from its inverse, but also between ‘similar’ relation types.

## Baseline Temporal Relation Classifier

Since the best-performing systems for temporal relation classification for both the news and clinical domains are learning-based, we will employ a learning-based system as our baseline. Below we describe how we train this baseline.

Without loss of generality, assume that (*e*_1_,*e*_2_) is an event–event/event–time pair such that (i) *e*_1_ precedes *e*_2_ in the associated text and (ii) (*e*_1_,*e*_2_) belongs to one of the 12 i2b2 temporal relation types. We create one training instance for each event–event/event–time pair in a training document that satisfies the two conditions above, labeling it with the relation type that exists between *e*_1_ and *e*_2_.

To build a strong baseline, we represent each instance using 167 features modeled after the top-performing temporal relation classification systems on TimeBank (e.g. 6–8) and the i2b2 corpus (e.g. 9, 10), as well as those in the TempEval shared tasks (e.g. 11–14). These features can be divided into six categories, as described below.

**Lexical (17 features).** Word unigrams, bigrams and trigrams formed from the context within a window of two words surrounding *e*_1_/*e*_2_, the strings and the head words of *e*_1_ and *e*_2_, and whether *e*_1_ and *e*_2_ have the same string.
**Grammatical (133 features).** The POS tags of the head words of *e*_1_ and *e*_2_, the POS tags of the five tokens preceding and following *e*_1_ and *e*_2_, the POS bigram formed from the head word of *e*_1_/*e*_2_ and its preceding token, the POS tag pair formed from the head words of *e*_1_ and *e*_2_, the prepositional lexeme of the prepositional phrase (PP) if *e*_1_/*e*_2_ is headed by a PP, the prepositional lexeme of the PP if *e*_1_/*e*_2_ is governed by a PP, the POS of the head of the VP if *e*_1_/*e*_2_ is governed by a VP, whether *e*_1_ syntactically dominates *e*_2_ (6), the shortest path from *e*_1_ to *e*_2_ in the associated syntactic parse tree, and four binary features as in D’Souza and Ng (7) encoding the grammatical roles of *e*_1_*/e*_2_ in their participating dependency relations of which there are 25 unique types automatically extracted from this dataset. We obtain POS tags, parse trees and dependency trees using the Stanford CoreNLP tool.^3^
**Entity attributes (8 features).** The type, modality and polarity of *e*_1_ and *e*_2_ if they are events (if one of them is a time expression, then its modality and polarity attributes will have the value NULL), pairwise features formed by pairing up the type and modality attribute values of *e*_1_/*e*_2_.
**Distance (2 features).** The distance between *e*_1_ and *e*_2_ in number of tokens, whether *e*_1_ and *e*_2_ in the same sentence.
**Semantic (4 features).** The subordinating temporal role token of *e*_1_/*e*_2_ if it appears within a temporal semantic role argument (12), and the first WordNet synset to which *e*_1_/*e*_2_ belongs.
**Section creation time (SCT) related (3 features).** The temporal relation type between *e*_1_/*e*_2_ and the creation time of the section in which it appears [its value can be one of the 3 relation types (i.e. **Before**, **After** or **Overlap**) or NULL if no relation exists], and whether *e*_1_ and *e*_2_ have different relation types with the SCT.

### Training specialized classifiers

After creating the training instances, we can train a temporal relation classifier on them using an off-the-shelf learner and use the resulting classifier to classify the test instances. However, Tang *et al.* (9), the best performer in the shared task, showed that performance can be improved by training four specialized classifiers rather than just one for classifying all temporal relation instances. Specifically, they trained two intra-sentence classifiers, one for classifying event–event pairs and the other event–time pairs. They also trained two inter-sentence classifiers, one for classifying coreferent event pairs and the other for classifying event pairs in neighboring sentences.

Since Tang *et al*.’s (9) approach looked promising, we integrated their four specialized classifiers into our machine learning framework in order to build a strong baseline. Below we describe Tang et al.’s method for creating instances for training and testing each of the four specialized classifiers.

**Training and applying an intra-sentence event–event classifier.** A naive way to create training/test instances would be to create one training/test instance from each pair of events. This, however, would create a training set with a skewed class distribution, as the negative (i.e. No-Relation) instances will significantly outnumber the instances that belong to one of the 12 relation types shown in Table 2. To address this problem, positive and negative training instances were created as follows. A positive instance was created from each event pair in which one of the 12 relation types exists, labeling the instance with the pertaining relation type. In addition, negative instances were created from two events only if (i) they were adjacent to each other (i.e. there was no intervening event); and (ii) no relation existed between them. During testing, test instances were created in the same way as the negative training instances.
**Training and applying an intra-sentence event–time classifier.** Training and test instances were created in the same way as in the event–event classifier.
**Training and applying an inter-sentence classifier for events in adjacent sentences.** The difficulty of temporal relation classification tends to increase with the distance between the elements in an event–event or event–time pair. Consequently, Tang *et al.* (9) considered event–event pairs only if the two elements involved in a pair are one sentence apart, ignoring event–time pairs entirely since very few of them have a temporal relation.

As mentioned before, one method for creating instances for training and testing would be to create one instance for each event–event/event–time pair. This method, however, skews the class distribution of the resulting dataset. Consequently, the following method was employed for creating training and test instances. A positive training instance was created from every event–event/event–time pair whose elements (1) had a temporal relation and (2) occurred in adjacent sentences, and by assigning a class value that was the relation type. In addition, a negative training instance was created from each pair of main events that appeared in adjacent sentences, where the main events of a sentence were simply the first and last events of a sentence. Test instances were created in the same way as the negative training instances.

**Training and applying an inter-sentence coreferent event classifier.** Unlike the previous classifier, this second inter-sentence classifier places no restriction on how far apart two events are. However, it handles only a subset of the inter-sentence temporal relations, namely those that are coreferent. The reason for this restriction is that it is intuitively easier to determine the relation type for two coreferent events, since they tend to overlap with each other.

A natural question is: how were two events posited as being coreferent? If two events had matching head words, they were naively grouped as coreferent.

Next, we describe how the instances for training and testing this inter-sentence coreferent event classifier were created. One training instance was created as a positive instance from every coreferent event pair in which a temporal relation exists, labeling it with the corresponding relation type. We could similarly create one negative training instance from every coreferent event pair that does not have any temporal relation. However, to reduce class skewness, following along Tang *et al.*’s method, negative training instances were created only from those coreferent event pairs where the two elements corresponded to main events. Test instances were created in the same way as the negative training instances.

In our experiments, we trained each of these four classifiers using SVM^multiclass^ (15). We tuned the regularization parameter, C, on the 20% of the training data that we reserved for development, and set the remaining learning parameters to their default values.^4^

## Our hybrid approach

In this section, we describe our hybrid learning-based and rule-based approach to temporal relation classification. The Six types of new features section describes our novel features, which will be used to augment the baseline feature set (see the Baseline temporal relation classifier section) to train each of the four specialized classifiers mentioned above. The Manual rule creation section outlines our manual rule creation process. The Combining rules and machine learning section discusses how we combine our hand-crafted rules and the learned classifiers.

### Six types of new features

#### Pairwise features

Recall that some of the features in the baseline feature set are computed based on either e_1_ or e_2_ but not both. Since our task is to predict the ‘relation’ between them, we hypothesize that ‘pairwise’ features, which are computed based on both elements, could better capture their relationship.

Specifically, we introduce pairwise versions of the head word feature and the two prepositional lexeme-based features in the baseline. In addition, we create one quadruple-wise feature by pairing up the type and modality attribute values of *e*_1_ with those of *e*_2_. Next, we create two ‘trace’ features, one based on prepositions and the other on verbs, since prepositions and verb tense have been shown to play an important role in temporal relation classification. The ‘preposition trace’ feature is computed by (i) collecting the list of prepositions along the path from *e*_1_/*e*_2_ to the root of its syntactic parse tree and (ii) concatenating the resulting lists computed from *e*_1_ and *e*_2_. The ‘verb trace’ feature is computed in a similar manner, except that we collect the POS tags of the verbs appearing in the corresponding paths.

#### Webster relations

Some events are not connected by a dependency relation but by a ‘lexical’ relation. We hypothesize that some lexical relations could be useful for temporal relation classification. Consider the following example:

(4) Her amylase was *mildly elevated* but has been *down* since then.

In this sentence, the two events, mildly elevated and down, are connected by an antonym relation. Statistically speaking, if (i) two events are in two clauses connected by the coordinating conjunction but, (ii) one is an antonym of the other and (iii) there is a temporal relation between them, then not only can we infer that they do not have any temporal overlap, but also it is likely that they have an asynchronous relation such as **Before** or **After**.

Given the potential usefulness of lexical relations for temporal relation classification, we create features based on four types of lexical relations present in Webster’s online thesaurus,^5^ namely synonyms, related-words, near-antonyms and antonyms. Specifically, for each event *e* appearing in the i2b2 corpus, we first use the head word of *e* to retrieve four lists, which are the lists corresponding to the synonyms, related words, near-antonyms and antonyms of *e*. Then, given a training/test instance involving *e*_1_ and *e*_2_, we create eight binary features: whether *e*_1_ appears in *e*_2_’s list of synonyms/related words/near- antonyms/antonyms, and whether *e*_2_ appears in *e*_1_’s list of synonyms/related words/near-antonyms/antonyms.

#### WordNet relations

Previous uses of WordNet for temporal relation classification are limited to synsets (e.g. 12). We hypothesize that other WordNet lexical relations could also be useful for the task. Specifically, we employ four types of WordNet relations, namely hypernyms, hyponyms, troponyms and similar, to create eight binary features for temporal relation classification. These eight features are created from the four WordNet relations in the same way as the eight features were created from the four Webster relations mentioned above.

#### Predicate–argument relations

So far we have exploited lexical and dependency relations for temporal relation classification. We hypothesize that semantic relations, in particular predicate–argument relations, could be useful for the task.

Consider the following example:

   (5) She was *discharged* to *rehab*.

Using SENNA (16), a PropBank-style semantic role labeler, we know that the CLINICAL_DEPARTMENT event rehab is the A4 argument of the OCCURRENCE event discharged. Recall that A4 is the end/destination point. Hence, we can infer that there is a **Begins** relation between the OCCURRENCE event and the CLINICAL_ DEPARTMENT event since the OCCURRENCE event begins at the end point.

Given the potential usefulness of relations between a predicate and its ‘numbered’ arguments (e.g. A0, A1, …) for temporal relation classification, we create one binary feature for each pairing of a numbered argument and a predicate, setting its value to 1 if according to SENNA *e*_1_ and *e*_2_ are in the predicate–argument relation specified by the pair.

To create additional features from predicate–argument relations, consider another PropBank-style predicate– argument relation type, cause. Assuming that *e*_2_ is in *e*_1_’s cause argument, we can infer that *e*_2_ occurs **Before** *e*_1_, since intuitively the cause of an action precedes the action.

Consequently, we create additional features for temporal relation classification based on four types of predicate–argument relations provided by SENNA, namely directional, manner, temporal and cause. Specifically, we create four binary features that encode whether argument *e*_2_ is related to predicate *e*_1_ through the four types of relations, and another four binary features that encode whether argument *e*_1_ is related to predicate *e*_2_ through the four types of relations.

#### Discourse relations

Rhetorical relations such as causation, elaboration and enablement could aid in tracking the temporal progression of the discourse (17). Hence, unlike syntactic dependencies and predicate–argument relations through which we can identify ‘intra-sentential’ temporal relations, discourse relations can potentially be exploited to discover both ‘inter-sentential’ and ‘intra-sentential’ temporal relations. However, no recent work has attempted to use discourse relations for temporal relation classification. In this subsection, we examine whether we can improve a temporal relation identifier via explicit and implicit PDTB-style discourse relations automatically extracted by Lin et al.’s (18) end-to-end discourse parser.

Let us first review PDTB-style discourse relations. Each relation is represented by a triple (*Arg1*, *sense*, *Arg2*), where *Arg1* and *Arg2* are its two arguments and sense is its sense/type. A discourse relation can be explicit or implicit. An explicit relation is triggered by a discourse connective. On the other hand, an implicit relation is not triggered by a discourse connective, and may exist only between two consecutive sentences. Generally, implicit relations are much harder to identify than their explicit counterparts.

Next, to motivate why discourse relations can be useful for temporal relation classification, we use two examples (see Table 3), one involving an implicit relation (Example (6)) and the other an explicit relation (Example (7)). For convenience, both sentences are also annotated using Lin *et al*.’s (18) discourse parser, which marks up the two arguments with the _Arg1 and _Arg2 tags and outputs the relation sense next to the beginning of Arg2.

**Table 3.** Examples illustrating the usefulness of discourse relations for temporal relation classification

(6)	{_Arg1 # Hypotension: per referral form. _Arg1} {_Arg2_RESTATEMENT Initially concern for sepsis in the setting of fevers and high blood count. _Arg2}
(7)	{_Arg1 At *operation*, there was no gross adenopathy, and it was felt that the tumor was completely excised. _Arg1} {_Arg2 The patient {_Conn_ASYNCHRONOUS thereafter _Conn} had a *benign convalescence*._Arg2}

The two arguments of each discourse relation, Arg1 and Arg2, are enclosed in curly brackets, and the sense of the relation is annotated.

In (6), we aim to determine the temporal relation between two PROBLEM events, hypotension and sepsis. The parser determines that a RESTATEMENT implicit relation exists between the two sentences. Intuitively, two temporally linked PROBLEM events within different discourse units connected by the RESTATEMENT relation implies some sort of synchronicity in their temporal relation. This means that the relation type is likely to be **Overlap** or **Simultaneous**. In this case, we can rule out Simultaneous: by definition, two non-coreferent events of the same type (e.g. hypotension and sepsis) cannot have a **Simultaneous** relation.

In (7), we aim to determine the relation between the TREATMENT event operation and the OCCURRENCE event benign convalescence. The parser determines that a ASYNCHRONOUS explicit relation triggered by thereafter exists between the two sentences, which in turn suggests that the two events are likely to have an asynchronous temporal relation such as **Before** or **After**. By considering just the discourse connective thereafter, we can infer that the correct temporal relation is **Before**.

Given the potential usefulness of discourse relations for temporal relation classification, we create four features based on discourse relations. In the first feature, if *e*_1_ is in Arg1, *e*_2_ is in Arg2 and Arg1 and Arg2 possess an explicit relation with sense *s*, then its feature value is *s*; otherwise its value is NULL. In the second feature, if *e*_2_ is in Arg1, *e*_1_ is in Arg2 and Arg1 and Arg2 possess an explicit relation with sense *s*, then its feature value is *s*; otherwise its value is NULL. The third and fourth features are computed in the same way as the first two features, except that they are computed over implicit rather than explicit relations.

#### Medical semantic relations

So far we have discussed two types of semantic relations: predicate–argument relations derived from PropBank and domain-independent relations derived from WordNet and Webster. These domain-independent semantic relations are by no means sufficient for classifying the temporal relations between events in the medical domain (henceforth ‘medical semantic relations’). For example, recall from Table 1 that two of the event types are PROBLEM and TREATMENT. It is not uncommon for PROBLEM and TREATMENT events to be denoted by medical names that do not appear in WordNet and Webster, so these general-purpose dictionaries may not provide any information about such events. Moreover, predicate–argument relations may not be useful for determining the temporal relation between a PROBLEM event and a TREATMENT event either, since a PROBLEM event is rarely an argument of a TREATMENT event and vice versa.

On the other hand, we hypothesize that medical semantic relations could sometimes provide useful information for temporal relation classification for cases that cannot be handled by domain-independent relations. Consider the following example:

(8) In the Amanda *the patient’s pain* resolved with *NTG* and morphine.

In Sentence (8), a medical semantic relation of type ‘treatment improves medical problem’ (TrIP) exists between PROBLEM event ‘the patient’s pain’ and TREATMENT event ‘NTG’. Since the treatment improves the problem, we can infer that NTG is **Ended_By** the patient’s pain. Note that no semantic relations can be extracted between these two events from WordNet and Webster, since NTG does not exist in these dictionaries. Predicate–argument information could be useful, but only if we combine the information from two predicate– argument relations, the relation between ‘resolve’ and ‘the patient’s pain’ and the relation between ‘resolve’ and ‘NTG’. On the other hand, medical semantic relations take into account the two events as well as the governing verb ‘at the same time’, thus providing us with information that cannot be directly inferred from predicate–argument relations.

Since there does not exist publicly available database that provides medical semantic relations, we develop a system for classifying such relations with the ultimate goal of employing them for temporal relation classification. Given that a corpus annotated with medical semantic relations exists, we adopt a corpus-based approach to building a medical semantic relation classification system. In the rest of this subsection, we introduce this corpus and describe our approach to medical semantic relation classification.

##### 2010 i2b2/VA challenge corpus

In 2010, i2b2 together with VA Salt Lake City Health Care System organized a community-wide shared task (19) that had as one of its subtasks to automatically classify TREATMENT events with PROBLEM events, TEST events with PROBLEM events and event pairs each of type PROBLEM into one of a set of predefined semantic medical relations. To facilitate system development and evaluation, the shared task organizers provided annotated data. This corpus now available to the general research community comprises 426 de-identified discharge summaries pre-partitioned into a training set (170 summaries) and a test set (256 summaries). It is worth noting that while the corpora used in the 2010 and 2012 i2b2 Challenges both comprise discharge summaries, they are distinct from each other.

In each summary in the 2010 corpus, the events and their ‘medical semantic relations’ are marked up. Unlike the 2012 Challenge, which considers six event types, the 2010 Challenge only considers three of these six types, namely TEST, PROBLEM and TREATMENT. In other words, the only semantic relations annotated are those that exist between events belonging to these three event types. In addition, unlike the 2012 Challenge, only intra-sentential semantic relations are annotated. There is one type of annotation that exist in the 2010 corpus but not the 2012 corpus, however: in addition to its type, a PROBLEM event has a second attribute, ‘assertion’, which conveys whether the problem was present, absent or possible in the patient, conditionally present in the patient under certain circumstances, hypothetically present in the patient at some future point, or mentioned in the patient report but associated with someone other than the patient. We will make use of this attribute in building our semantic relation classification system.

Eleven types of relations are annotated in the 2010 i2b2 corpus. A brief description of these relation types and the relevant statistics are provided in Table 4. As we can see from the table, the 11 relation types can be organized under five broader functional categories: (i) causal—wherein an event effects a change in another event; (ii) indicative—wherein an event indicates another event; (iii) demonstrative—wherein an event shows the existence or truth of another event by giving proof or evidence; (iv) generally informative—general information about the paired events and (v) no relations. Note that while there are 11 relation types, 3 of them denote the absence of a semantic relation between the corresponding events. The purpose of having ‘no relations’ is to ensure that every pair of TEST/PROBLEM/TREATMENT event is annotated, whether or not a semantic relation exists between them.

**Table 4.** The 11 unique relation types for medical semantic relation classification

Id	Relation	Example	Total (%)
**A**	**Causal relations**		
1	TrIP: Treatment improves medical problem	*Her pain* resolved after *surgery*	203 (0.6)
2	TrWP: Treatment worsens medical problem	Treated with *Zofran* with *no relief*	133 (0.4)
3	TrCP: Treatment causes medical problem	*Transdermal nitroglycerin* caused *headache*	526 (1.8)
**B**	**Indicative relation**		
4	PIP: Medical problem indicates medical problem	With *a moderate-sized, dense, fixed inferior defect* indicative of *scar*	2202 (7.5)
**C**	**Demonstrative relation**		
5	TeRP: Test reveals medical problem	*A postoperative MRI* revealed no *remarkable findings*	3051 (10.4)
**D**	**Generally information relations**		
6	TrAP: Treatment is administered for medical problem	start on *Decadron* 4 mg q6 to prevent *swelling*	2613 (8.9)
7	TrNAP: Treatment is not administered because of medical problem	*His Avandia* was discontinued secondary to *the side effect profile*	174 (0.6)
8	TeCP: Test conducted to investigate medical problem	*An ultrasound* was done to rule out *cholestasis*	504 (1.7)
**E**	**No relations**		
9	NTrP: No relation between treatment and problem	With *sutures* intact and no *erythema* or purulence noted	4462 (15.2)
10	NTeP: No relation between test and problem	Through the stay *his laboratories* remained normal and *his pain* controlled	2964 (10.1)
11	NPP: No relation between paired medical problems	He is somewhat *cantankerous* and *demanding* of the nurses	12503 (42.6)

Each relation type is defined on an ordered pair where concepts in the pair are as specified by the relation. The ‘Total’ and ‘%’ columns show the number and percentage of instances annotated with the corresponding relation type over all 426 reports, respectively.

Each semantic relation defined on an event–pair has the following characteristics: (i) has a *type*; (ii) is defined only on intra-sentence TREATMENT– PROBLEM, TEST–PROBLEM and PROBLEM– PROBLEM events pairs and (iii) is commutative in nature, in other words, a relation applicable to event pair (*e*_1typeX_, *e*_2typeY_), is also applicable when the pair is reversed (*e*_2typeY_, *e*_1typeX_).

##### Additional motivating examples

At the beginning of this subsection, we have provided an example illustrating why **TrCP** (a type of causal relations) could be useful for temporal relation classification. Below we use four examples to illustrate why the remaining four broader categories of semantic relation types shown in Table 4, including ‘no relations’, may also be useful for temporal relation classification.

(9) Patient returns from the nursing home with *fever*, *leukocytosis* and azotemia.
(10) Residual deficits include right side facial droop from previous stroke and *anterior aphasia* with *word finding difficulties*.
(11) The patient had *bone marrow biopsy*, August, 2003, for *persistent pancytopenia*.
(12) *Her creatinine* continued to increase, likely due to *her worsening liver failure*.

First, consider Sentence (9), which shows that the absence of a medical semantic relation between two events could also be useful for temporal relation classification. In this sentence, the two PROBLEM events ‘fever’ and ‘leukocytosis’ are part of a comma-separated series. Intuitively, the most likely temporal relations between them are **Overlap** and **Simultaneous**. Given they are not related to each other (via any medical semantic relation), it is unlikely that they occur simultaneously. Hence, we can infer that the two events **Overlap** in time.

Sentence (10) shows why ‘indicative relations’ could be useful for temporal relation classification. In this sentence, there is a prepositional dependency triggered by the preposition with between the two PROBLEM events, ‘anterior aphasia’ and ‘word finding difficulties’. Intuitively, the most likely temporal relations between them are **Overlap** and **Simultaneous**. Since there is a **PIP** relation between them (i.e. ‘aphasia’ indicates ‘word finding difficulties’), it is likely that the two problems occur simultaneously. Also note that the knowledge of aphasia indicating word finding difficulties in people is domain-specific, and hence such knowledge cannot be obtained from an open-domain lexical resource.

Sentence (11) shows why generally informative relations could be useful for temporal relation classification. In this sentence, there is a **TeCP** relation between the TEST event ‘bone marrow biopsy’ and the PROBLEM event ‘persistent pancytopenia’, meaning that bone marrow biopsy is conducted to investigate persistent pancytopenia. Intuitively, for a test conducted to investigate a problem, the problem should already be present. In other words, we can infer that a TEST conducted to investigate a PROBLEM overlaps with but happens after the PROBLEM, so there is a temporal **Overlap_After** relation between the events.

Sentence (12) shows why ‘demonstrative relations’ could be useful for temporal relation classification. In this sentence, the presence of the phrase ‘due to’ allows us to infer that the temporal relation between TEST event ‘Her creatinine’ and PROBLEM event ‘her worsening liver failure’ is either **After** or **Overlap_After**. Making use of the fact that there is a **TeRP** relation between the TEST and the PROBLEM (i.e. the TEST reveals the PROBLEM), the PROBLEM continues to exist after the TEST. Hence, we can infer that the correct temporal relation between them is **Overlap_After**.

##### Medical semantic relation classification

In this subsection, we describe our corpus-based approach to medical semantic relation classification. We adopt an ‘ensemble’ approach, where a classification decision is made by combining the output of multiple classifiers. Our decision to employ an ensemble approach is motivated by the following observations. First, the best-performing system (20) employs an Support Vector Machine (SVM) classifier trained on a set of ‘flat features’ (i.e. features that are either discrete- or real-valued). More recently, however, Zhu *et al*. (21) have obtained better results by employing a tree kernel-based approach. We hypothesize that classification performance can be further improved by combining these and other classifiers. Specifically, in addition to training SVM classifiers that employ flat features (as in Rink *et al.* (20)) and structured features (as in Zhu *et al.*’s tree kernel-based approach), we include in our ensemble a *k*-nearest-neighbor classifier. Below we describe the implementation details of these three classifiers.

(1) **SVM classifier with flat features**
We create training instances for the flat-feature SVM classifier as follows. First, we form training instances between every pair of (PROBLEM, TEST and TREATMENT) events in the training documents, labeling an instance with its relation type. Since the instances belonging to the three ‘no relation’ classes significantly outnumber those belonging to the remaining eight classes, we reduce data skewness by downsampling instances belonging to the three ‘no relation’ classes.^6^
Each instance is represented using 37 feature types modeled according to Rink *et al.*’s (20) features. These 37 feature types can be broadly divided into five categories:
  **Context (13 groups).** The words, the POS tags, the bigrams, the string of words, the sequence of phrase chunk types and the concept types used between *e*_1_ and *e*_2_; the word preceding *e*_1_/*e*_2_; any of the three words succeeding *e*_1_/*e*_2_; the predicates associated with both concepts and a feature that indicates whether a conjunction regular expression matched the string of words between *e*_1_ and *e*_2_.
  **Similarity (5 groups).** We find the concept pairs in the training set that are most similar to the (*e*_1_,*e*_2_) pair (i.e. its nearest neighbors), and create features that encode the statistics collected from these nearest neighbors. To find the nearest neighbors, we (1) represent each pair in the training set as a sequence; (2) define the number of nearest neighbors to use and (3) define a similarity metric to compute the similarity of two sequences.
  Following Rink *et al*. (20), we employ five methods to represent a pair. The five methods are: (i) as a sequence of POS tags for the entire sentence containing the pair; (ii) as a phrase chunk sequence between the two events; (iii) as a word lemma sequence beginning the two words before the first event, up to and including the second word following the second event in the pair; (iv) as an event type sequence for all the events found in the sentence containing the pair and (v) as a shortest dependency path sequence connecting the two events. Table 5 shows an example of these five ways of generating sequences from the TEST event ‘her exam’ and PROBLEM event ‘her hyperreflexia’ in the sentence ‘Postop, her exam only improved slightly in her hyperreflexia’. Note that for better generalization, the two events are replaced with their event type (i.e. her exam and her hyperreflexia are replaced with test_e1_ and problem_e2,_ respectively) before sequence generation. We use the Levenshtein distance (22) as the similarity metric.

**Table 5.** Examples of the five ways of sequence generation

Generation method	Sequence
(1)	RB VB, test_e1_ RB VBD RB IN problem_e2_
(2)	ADVP VP ADVP PP
(3)	postop, test_e1_ only improve slightly in problem_e2_
(4)	test_e1_ problem_e2_
(5)	test_e1_—nsubj → prep ← pobj—problem_e2_
  After finding the nearest neighbors for each of the five methods of sequence representation, we create features as follows. For each method, we compute the percentage of nearest neighbors belonging to each of the 11 relation types, and then create 11 features whose values are these 11 numbers.
  **Single concept (11 groups**)**.** Any word lemma from *e*_1_/*e*_2_; any word used to describe *e*_1_/*e*_2_; the event type for *e*_1_/*e*_2_; the string of words in *e*_1_/*e*_2_; the concatenation of assertion types for both concepts and the sentiment category (i.e. positive or negative) of *e*_1_/*e*_2_ obtained from the General Inquirer lexicon (23).
  **Wikipedia (6 groups).** Six features are computed based on the Wikipedia articles, their categories and the links between them. The first feature encodes whether neither *e*_1_ nor *e*_2_ contains any substring that may be matched against the title of an article. The second feature encodes whether the links between the articles retrieved based on the two events are absent. The next two features encode whether a link exists from the article pertaining to *e*_1_ (*e*_2_) to the article pertaining to *e*_2_ (*e*_1_). The fifth feature encodes whether there are links between the articles pertaining to both concepts. The last feature encodes whether both concepts have the same concept type according to their Wikipedia categories.
  **Vicinity (2 groups).** The concatenation of the type of *e*_1_ and the type of the closest event preceding *e*_1_; and the concatenation of the type of *e*_2_ and the type of the closest event succeeding *e*_2_.
  After creating the training instances, we train a 11-class classifier on them using SVM^multiclass^ (15). We set C, the regularization parameter, to 10 000, since preliminary experiments indicate that preferring generalization to overfitting (by setting C to a small value) tends to yield poorer classification performance. The remaining learning parameters are set to their default values. We then use it to make predictions on the test instances, which are generated in the same way as the training instances.
(2) **SVM classifier with structured features**
  Within this framework, each instance is represented using a single structured feature computed from the parse tree of the sentence containing the event pair. Since publicly available SVM learners capable of handling structured features can only make binary predictions, we train 11 SVM classifiers, one for representing each semantic relation, where in each classifier’s training data, a positive instance is one whose class value matches the semantic relation class value of the classifier, and a negative instance is one with other class values applicable to the given event pair. To reduce data skewness, we determine the optimal ratio of positive to negative instances on held-out development data, which is composed of 30 randomly chosen training documents.^7^ We set C, the regularization parameter, to 100 based on the development data.
  While we want to use a parse tree directly as a feature for representing an instance, we do not want to use the entire parse tree as a feature. Specifically, while using the entire parse tree enables a richer representation of the syntactic context of the two events than using a ‘partial’ parse tree, the increased complexity of the tree also makes it more difficult for the SVM learner to make generalizations.
  To strike a better balance between having a rich representation of the context and improving the learner’s ability to generalize, we extract a subtree from a parse tree and use it as the value of the structured feature of an instance. Specifically, given two events in an instance, and the associated syntactic parse tree *T*, we retain as our subtree the portion of *T* that covers (i) all the nodes lying on the shortest path between the two entities and (ii) all the immediate children of these nodes that are not the leaves of *T*.
  This subtree is known as a ‘simple expansion tree’, and was first used by Yang *et al*. (24) as a structured feature for the pronoun resolution task. Note that some of the flat features, including the event type attribute, are not encoded in the simple expansion tree. Hence, we encode these attribute values in the tree as follows: we replace the non-terminal symbol of the tree node that spans each of the two entities under consideration with its event attribute values. To better understand how a simple expansion tree is computed, we show in Figure 1 the simple expansion tree created for the relation **TeRP** between TEST event ‘evaluated’ and PROBLEM event ‘desaturate’. Note that all and only those terminal and non-terminal nodes that are circled or squared are part of the tree.^8^

**Figure 1.**
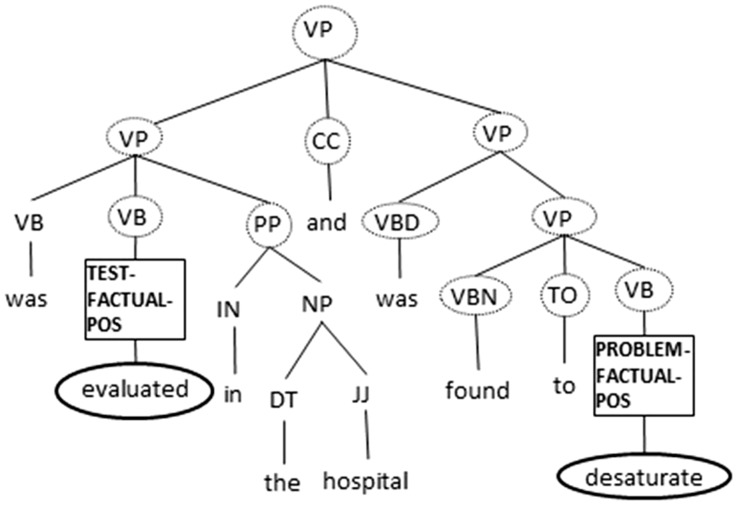
Example of a simple expansion tree.
  After training the 11 tree kernel-based semantic relation classifiers, we can apply them to classify a test instance. The class value of an instance is determined based on the classifier with the maximum classification confidence, where the classification value of an instance is its signed distance from the SVM hyperplane.
(3) ***K*-nearest neighbor classifier**
  Recall that we employed five ways to represent an event pair when creating the nearest-neighbor features in our flat-feature classifier. In the process, we observed that the nearest neighbors obtained using the third way of sequence generation (where we represent a pair as a sequence of word lemmas) are quite indicative of the correct semantic relation class. Motivated by this observation, we employ as the third classifier in our ensemble a *K*-nearest-neighbor classifier, where each event pair is represented using the third way of sequence generation. Like before, we employ the Levenshtein distance metric to measure similarity. Unlike before, however, we set the number of nearest neighbors to 1.
(4) **The ensemble**
  Given a test instance, we determine its medical semantic relation type by combining the outputs of the ‘flat-feature’ classifier, the ‘structured-feature’ classifier and the ‘K-nearest-neighbor’ classifier as follows.
  First, we derive from the output of each classifier an 11-element probability vector, which encodes the probability that the given test instance belongs to each of the 11 relation types. Specifically, for the flat-feature classifier and the structured-feature classifier, the SVM outputs a confidence value for each class. To obtain the probability vector for each of these classifiers, we first normalize the confidence value associated with each class so that it falls within the [0,1] range, and then normalize the resulting values so that they sum to 1. For the *k*-nearest-neighbor classifier, we generate the probability vector by tallying the votes of the 200 nearest neighbors. Note that we give a higher weight to the vote cast by the nearest neighbor. More specifically, we assign a weight of 0.5 to the vote cast by the nearest neighbor, and a weight of 0.5/199 to the vote cast by each of the remaining 199 nearest neighbors.
  Next, we combine the three probability vectors as follows:
  (1) $$C_{\text{combined}}=0.\text{4}\times P_{\text{tree}}+0.\text{5}\times P_{\text{flat}}+0.\text{1}\times P_{\text{knn}},$$
  where the three combination weights are determined using held-out development data. The class that we assign to the test instance is simply the class having the maximum value according to *C*_combined_.

##### **Evaluating** **semantic relation classification**

To get an idea of how well our ensemble approach to medical semantic relation classification is and whether the ensemble indeed performs better than the three classifiers it relies on. Following the 2010 i2b2/VA evaluation scheme, a semantic relation classification system is evaluated on all but the ‘no relation’ types. In other words, even if the classifier classifies an instance correctly into any of the relations **NTrP**, **NTeP** and **NPP** they are not counted.

**Dataset.** As mentioned before, we use the 170 training documents from the 2010 i2b2/VA corpus for classifier training, and reserve the 256 test documents for evaluating system performance.
**Evaluation metrics.** Results are reported in terms of *micro F-score* (see Sebastiani (25) for its definitions).
**Results and discussion.** Table 6 shows the medical semantic relation classification results. Again, results are computed over all but the no relation types. The first column corresponds to the type of the classifier, and the second column shows the micro *F*-score for each classifier.

**Table 6.** Semantic relation classification results

	Classifiers	*F* score
1	Tree	59.6
2	Knn	61.4
3	Flat	61.6
4	Ensemble	66.9

The best performing system is the ensemble system, which achieves a micro *F*-score of 66.9%. This translates to a relative error reduction of 10–18% over the tree- feature classifier, 8–14% over the knn classifier and 14% over the flat-features classifier.^9^

##### Using medical semantic relations for temporal relation classification

Given the potential usefulness of medical semantic relations for temporal relation classification, as demonstrated earlier through Examples (8)–(12), we create features for temporal relation classification based on these semantic relations. One feature encodes the relation type predicted by our ensemble-based medical semantic relation classification system. The remaining features come from the 36 feature groups that we used for medical semantic relation classification. Note that these features are only applicable to inter-sentential TREATMENT–PROBLEM, TEST–PROBLEM and PROBLEM–PROBLEM event pairs, since our semantic relation classifiers were trained on these three types of event pairs.

### Manual rule creation

As noted before, we adopt a hybrid learning-based and rule-based approach to temporal relation classification. Hence, in addition to training a temporal relation classifier, we manually design a set of rules in which each rule returns a temporal relation type for a given test instance. We hypothesize that a rule-based approach can complement a purely learning-based approach, since a human can combine the available features into rules using commonsense knowledge that may not be accessible to a learning algorithm.

The design of the rules is partly based on intuition and partly data driven: we first use our intuition to come up with a rule and then manually refine it based on the observations we made on the i2b2 training documents. Note that the test documents are reserved for evaluating final system performance. We order these rules in decreasing order of accuracy, where the accuracy of a rule is defined as the number of times the rule yields the correct temporal relation type divided by the number of times it is applied, as measured on the training documents. A new instance is classified using the first applicable rule in the ruleset.

Some of these rules were shown in the Six types of new features section when we motivated each feature type with examples as well as in Appendix A. Our final ruleset can be accessed via a web link (see Footnote 2).

### Combining rules and machine learning

We investigate two ways to combine the hand-crafted rules and the machine-learned classifiers.

In the first method, we employ all of the rules as additional features for training each of the four specialized classifiers. The value of each such feature is the temporal relation type predicted by the corresponding rule.

The second method can be viewed as an extension of the first one. Given a test instance, we first apply to it the ruleset composed only of rules that are at least 75% accurate. If none of the rules is applicable, we classify it using one of the four classifiers employed in the first method.^10^

## Evaluation: the first setting

### Experimental setup

In this section, we will conduct experiments under the first setting, where we assume we are given gold-standard temporal relations (i.e. each instance belongs to one of the 12 relations).

**Dataset.** As mentioned before, we use the 190 training documents from the i2b2 corpus for classifier training and manual rule development and reserve the 120 test documents for evaluating system performance.
**Evaluation metrics.** We employ micro *F-*score (*F*^mi^) and macro F-score (*F*^ma^) to evaluate our 12-class temporal relations classifier.^11^

### Results and discussion

Table 7 shows the results for our 12-class temporal relation classification task when the experiments are conducted under the first setting (see the Introduction section), where gold-standard temporal relations are used. The five columns of the results tables correspond to five different system architectures. The ‘Features’ column corresponds to a purely learning-based system where the results are obtained simply by training a temporal relation classifier using the available features. The next two columns correspond to two purely rule-based systems, differing by whether all rules are used regardless of their accuracy or whether only high-accuracy rules (i.e. those that are at least 75% accurate) are used. The rightmost two columns correspond to the two ways of combining rules and machine learning described in the Combining rules and machine learning section.

**Table 7.** 12-Class micro and macro *F* scores of classifying gold-standard temporal relations as features are added incrementally to the baseline

		Features		All rules		All rules with accuracy ≥0.75		Features + rules as features		Rules + features + rules as features	
	Feature type	F^mi^	F^ma^	F^mi^	F^ma^	F^mi^	F^ma^	F^mi^	F^ma^	F^mi^	F^ma^
1	Baseline	55.4	50.9	–	–	–	–	–	–	–	–
2	+ Pairwise	55.5	51.2	40.0	31.9	16.2	23.3	57.4	53.1	58.1	53.0
3	+ WordNet	55.6	51.1	40.0	31.9	16.2	23.3	57.2	53.0	57.9	52.9
4	+ Webster	55.8	51.3	40.0	31.9	16.2	23.3	57.3	53.0	58.0	52.9
5	+ PropBank	55.8	51.3	45.4	44.7	21.3	34.7	57.6	53.1	59.7	57.7
6	+ Discourse	56.2	51.5	47.3	47.8	24.0	39.2	57.9	53.2	61.1	60.7
7	+ Medical relations	56.6	53.0	47.7	49.6	26.2	39.8	57.7	54.4	**62.9**	**62.5**

The strongest results are boldfaced.

On the other hand, the rows of the tables differ in terms of what features are available to a system. In row 1, only the baseline features are available. In the subsequent rows, the six types of features discussed in the Our hybrid approach section are added incrementally to the baseline feature set. So, the last row corresponds to the case where all feature types are used.

A point merits clarification. It may not be immediately clear how to interpret the results under, for instance, the ‘All Rules’ column. In other words, it may not be clear what it means to add the six types of features incrementally to a rule-based system. Recall that one of our goals is to compare a purely learning-based system with a purely rule-based system, since we hypothesized that humans may be better at combining the available features to form rules than a learning algorithm. To facilitate this comparison, all and only those features that are available to a learning-based system in a given row can be used in hand-crafting the rules of the rule-based system in the same row. The other columns involving the use of rules can be interpreted similarly.

The best-performing system architecture is the hybrid architecture where high-accuracy rules are first applied and then the learned classifier is used to classify those cases that cannot be handled by the rules (see the rightmost column of Table 7). When all the features are used in combination with this architecture, the system achieves a micro *F*-score of 62.9% and a macro *F*-score of 62.5%. This translates to a relative error reduction of 17–24% in comparison to the baseline result shown in row 1. Regarding the usefulness of each of the seven types of features in this best-performing architecture, we found that adding pairwise features, predicate–argument relations, discourse relations and medical semantic relations significantly improves both micro and macro *F*-scores.^12^ The dependency, WordNet and Webster relations are not useful.^13^

Among the remaining four architectures, the version of the rule-based system where only the high-accuracy rules are used performs the worst, owing to the low coverage of the ruleset. Comparing the ‘Features’ system and the ‘All Rules’ system, we see that ‘All Rules’ is always significantly worse than ‘Features’. These results suggest that overall, the machine learner is better at combining the available knowledge sources than the human for temporal relation classification. The question, however, is: does the machine learner make mistakes on different instances than the human? By comparing the results of the two feature-based systems, ‘Features’ and ‘Features + Rules as Features’, we can infer that the answer is yes. Since the latter is significantly better than the former, the incorporation of the hand-crafted rules into the feature set is beneficial for the learner. In other words, the use of rules as features helps fix some of the mistakes made by the learner.

## Temporal relation identification

In the previous section, we evaluated our approach under the first setting, where we assume we are given only instances that belong to one of the 12 relation types. Recall from the introduction that we make the task more challenging by also evaluating our approach under a second setting, where we assume the instances we are given may or may not belong to one of the 12 relation types. For the second setting, we adopt a ‘pipeline’ system architecture where we first employ a relation ‘identification’ system to determine whether a test instance possesses a temporal relation. We then use the relation ‘classification’ system described in the Our hybrid approach section to classify only those instances the relation identification system determined possessed a temporal relation. The rest of this section describes our temporal relation identification system.

Given the success of our hybrid approach to relation classification, we employ a hybrid approach to relation identification. Specifically, given a test instance *i*, we first apply a set of hand-crafted rules to determine whether *i* has a relation. If *i* cannot be classified by any of the rules, we employ a learned identifier to determine whether *i* has a relation.

Two questions naturally arise. First, how can we design the hand-crafted rules? Second, how can we train a classifier for identifying relations? We answer these two questions in the next two paragraphs.

We design the hand-crafted identification rules as follows. As positive rules (i.e. rules that determine that an instance has a relation), we simply use all the rules that we hand-crafted for relation classification in the Manual rule creation section. To design ‘negative’ rules (i.e. rules that determine that an instance has no relation), we employ the same data-driven procedure that was used to design the relation classification rules (see the Manual rule creation section).

Next, we describe how to train a classifier for identifying temporal relations. We employ a natural way of creating training instances: we use all event–event and event–time pairs in the training set that have a relation as positive instances, and the remaining ones as negative instances. As before, rather than training just one classifier for identifying temporal relations, we train four specialized classifiers for identifying relations using the same division that we described in the Training specialized classifiers section. It is worth mentioning, however, that the negative instances significantly outnumber the positive ones, since most pairs do not have a relation. But since training on a dataset with a skewed class distribution may adversely affect the performance of a classifier, for each of the four specialized classifiers, we employ simple pruning heuristics to prune the negative training instances before training the classifier.^14^

The remaining question is: what features should we use to represent each training/test instance? We experimented with three options. The simplest option is to employ the same features that we used to train our classifiers for relation classification in the Our hybrid approach section. Note that many of these features are extracted from syntactic parse trees. Since it is not clear whether these features have adequately encoded all the useful information that we can possibly extract from a parse tree, perhaps the simpler thing to do, which we consider in our second option, is to employ just the syntactic parse tree containing the two entities involved in an instance.^15^ Recall that advanced machine learning algorithms such as SVMs have enabled a parse tree to be used as a ‘structured’ feature (i.e. a feature whose value is a linear or hierarchical structure, as opposed to a ‘flat’ feature, which has a discrete or real value), owing to their ability to employ ‘kernels’ to efficiently compute the similarity between two potentially complex structures. In particular, given two parse trees, we compute their similarity using a convolution tree kernel (26).

As in medical semantic relation classification, we employ a simple extraction tree as a structured feature to represent an instance for temporal relation identification. Recall that a simple expansion tree is the portion of a parse tree that covers (i) all the nodes lying on the shortest path between the two entities and (ii) all the immediate children of these nodes that are not the leaves of *T*. Some of the flat features employed in the first option, including the event attributes (i.e. type, polarity and modality) and the time attribute (i.e. type), are not encoded in the tree. As a result, we encode these attribute values in the tree as follows: we replace the parent node of each entity under consideration with its event/time attribute values.

Given that we employ flat features in our first option and a tree feature in our second option, a natural third option is to combine the flat and tree features to train a classifier. To compute the similarity between two instances containing both flat and tree features, we first compute the similarity of their flat features using a linear kernel and the similarity of their tree features using a tree kernel, and then combine these two kernels using a composite kernel.^16^

After training the four specialized classifiers, we can apply them to classify whether a test instance has a relation or not. By default, any instance whose classification value is at least 0 is classified as having a relation; otherwise, it is classified as having no relation. Note that the classification value of an instance is simply its signed distance from the SVM hyperplane.

Since we are using the relation identification system to filter the no relation instances prior to relation classification, the performance of the downstream relation classification system depends to a large extent on the performance of the identification system. If the identification system misclassifies many positive instances (as negative), it will harm the recall of the classification system; on the other hand, if it misclassifies many negative instances (as positive), it will harm the precision of the classification system.

Ideally, we want to optimize the performance of the identification classifier such that when it is used in combination with the ‘classification system’, the *F*-score of the classification system is maximized. However, the identification classifier is trained to maximize classification accuracy on identification. To maximize the *F*-score of the classification system instead, we propose to adjust the ‘classification threshold’ (i.e. the threshold that determines whether an instance should be classified as positive or not). Recall that currently we employ a classification threshold of 0, meaning that all and only those instances whose classification value is 0 or above are classified as positive. By adjusting this threshold, we can potentially vary the *F*-score of the classification system. Specifically, by lowering the threshold, more instances will be classified as positive, potentially improving the recall of the classification system. By the same token, increasing the threshold could improve its precision.

Given this observation, we tune the classification threshold to maximize the *F*-score of the classification system on the development set, which is composed of 20% of the training data. In other words, we first train both the classifiers for relation identification and classification on the remaining 80% of the training data. Then we obtain relation classification results on the development set by varying the classification thresholds applied to the relation identification classifiers (each of the four specialized identification classifiers will have its classification threshold tuned independently of the others).^17^ The thresholds that yield the best relation classification *F*-score on the development set are applied to obtain relation classification results on the test data.

Since our results are reported in terms of both micro and macro *F*-scores, we obtain thresholds that maximize macro *F* and those that maximize micro *F* separately.

## Evaluation: the second setting

Next, we conduct experiments under the second setting, where we obtain temporal relation classification results using automatically identified temporal relations.

Results, expressed in terms of micro and macro *F*, are shown in Table 8, where the rows and columns can be interpreted in the same manner as those in Table 7. As expected, the results obtained using automatically identified relations are significantly lower than those obtained using gold-standard temporal relations. Nevertheless, the same conclusions that we drew from the results in Table 7 are also applicable to the results in Table 8. It is worth mentioning, however, that the best-performing system is still the ‘Rules + features + rules as features’ architecture when used in combination with all the feature types, achieving a micro *F*-score of 31.7% and a macro *F*-score of 39.4%. This translates to a significant relative error reduction of 8–14% in comparison to the baseline.

**Table 8.** 12-class micro and macro *F*-scores of classifying automatically identified temporal relations as features are added incrementally to the baseline

		Features		All rules		All rules with accuracy ≥ 0.75		Features + rules as features		Rules + features + rules as features	
	Feature type	F^mi^	F^ma^	F^mi^	F^ma^	F^mi^	F^ma^	F^mi^	F^ma^	F^mi^	F^ma^
1	Baseline	26.2	30.1	–	–	–	–	–	–	–	–
2	+ Pairwise	26.5	30.6	17.1	18.9	9.9	14.3	26.7	31.4	27.7	33.0
3	+ WordNet	26.5	30.7	17.2	18.9	9.9	14.3	26.6	31.2	27.6	32.9
4	+ Webster	26.5	30.7	17.2	19.0	9.9	14.3	26.7	31.2	27.6	32.9
5	+ PropBank	26.5	30.8	21.2	29.3	15.4	24.4	26.8	31.3	29.1	36.7
6	+ Discourse	26.6	30.8	21.9	30.5	18.8	29.6	26.8	31.3	30.0	38.8
7	+ Medical relations	26.7	30.9	22.9	33.6	22.3	32.2	27.2	31.5	**31.7**	**39.4**

The strongest results are boldfaced.

## Evaluation: the I2B2 setting

### Experimental setup

Finally, by mapping each of the 12 classes from our classifier’s output in the second setting to their respective three class counterparts, we obtained 3-class classification results as per the 2012 i2b2 shared task.

**Dataset.** For classifier evaluation, test data annotations are taken from the dataset used in the shared task. These data comprise the same set of events, time expressions and the same temporally related event–event and event–time pairs, but with the relation annotations taken from the three class set instead.
**Evaluation metrics.** The *precision* (P), *recall* (R) and *F-score* (F) reported in this article are computed using the i2b2 shared task evaluation script. We use the default scoring scheme, where precision is defined as the total number of system output TLINKs that can be verified in the gold standard closure divided by the total number of system output TLINKs, and recall is the total number of gold standard output TLINKs that can be verified in the system closure divided by the total number of gold standard output TLINKs.

### Results and discussion

Results, reported in terms of precision, recall and *F*-score, are shown in Table 9. The rows and columns of the table can be interpreted in the same manner as Tables 7 and 8 wherein rows correspond to features used by the system and the columns reflect the different system architectures. It is noteworthy that even in this three-class setting, there are incremental improvements in classification performance on adding the new features, thus in turn showing that the features apart from being useful to the task of temporal relation classification are effective even at different granularities of the relations. Also, identical to the 12-class setting, the best performing system in this setting is the ‘Rules + features + rules as features’ hybrid system when all features are available to it. It achieves an *F*-score of 70.2. The best performer in the shared task, Tang *et al*. (9), reported an *F*-score of 69.3. Thus, even as a three-class classifier, our feature-rich hybrid system shows improvement over the state-of-the-art. In addition, we experimentally verify the complexity of the 12-class task versus the three-class task by comparing their respective *F*^mi^ scores using the same formula used for computing the scores reported in Tables 7 and 8. In the three-class setting, the best system achieves an *F*^mi^ score of 50.7; from Table 8 we see that the *F*^mi^ score of the same system as a 12-class classifier is much lower, 31.7. This tells us that the classifier makes more classification errors as a fine-grained classifier than when used for classifying the three broad relation types, thus in turn verifying that task complexity increases with finer granularity of the relations.

**Table 9.** 3-Class precision, recall and *f*-measure of classifying automatically identified temporal relations as features are added incrementally to the baseline

		Features			All rules			All rules with accuracy ≥0.75			Features + rules as Features			Rules + features + rules as features		
	Feature type	P	R	F	P	R	F	P	R	F	P	R	F	P	R	F
1	Baseline	75.1	62.9	68.5	–	–	–	–	–	–	–	–	–	–	–	–
2	+ Pairwise	79.7	60.6	68.9	62.1	57.0	59.4	91.0	37.4	53.0	79.6	61.0	69.1	76.7	62.3	68.7
3	+ WordNet	79.7	60.7	68.9	62.9	59.3	61.0	90.8	38.3	53.8	79.7	61.1	69.1	76.7	62.4	68.8
4	+ Webster	79.7	60.7	68.9	62.9	59.3	61.0	90.8	38.3	53.8	79.7	61.1	69.1	76.7	62.4	68.8
5	+ PropBank	79.8	60.7	69.0	63.3	61.3	62.3	90.8	40.2	55.8	79.6	61.3	69.2	77.2	63.1	69.4
6	+ Discourse	79.5	60.9	69.0	61.9	62.9	62.4	88.8	41.7	56.7	79.7	61.2	69.2	75.9	64.4	69.7
7	+ Medical relations	78.6	61.6	69.1	62.2	63.3	62.7	88.2	45.0	59.6	79.8	61.7	69.6	**76.1**	**65.1**	**70.2**

The strongest results are boldfaced.

## Error analysis

To gain additional insights into the errors made by the 12-class relation classification system and the relation identification system, we perform an error analysis of each of them.

### Relation classifications errors

We constructed the confusion matrix based on the gold standard and predicted relation types on the test set, and found that there are three types of confusions that account for nearly 72% of the classification errors. Below we illustrate each of these three types of confusions with examples.

**Simultaneous confused as overlap.** This is the most frequent source of confusion, accounting for 30.8% of the errors. The following example illustrates this confusion:
  *04-24 PICC Bld Cx: pseudomonas Diaz to zosyn, cipro, cefepime, Tardugno—staph epi- Gray to vanc*

In this sentence, the treatments ‘zosyn’, ‘ciporo’, ‘cefepime’ and ‘Tardugno’ are all given at the same time, and therefore are temporally **Simultaneous**. However, there are many cases where events separated by commas are overlapping rather than simultaneous. Determining whether the relation should be **Simultaneous** or **Overlap** requires an understanding of the nature of the events and cannot simply be inferred based on syntactic patterns. This poses a challenge to the relation identification system.

Recall that the i2b2 organizers grouped **Overlap** and **Simultaneous** under the same broad relation type. The fact that almost a third of our relation classification errors were related to confusion between **Overlap** and **Simultaneous** seems to be consistent with the notion that merging them was a wise decision. As Pustejovsky and Stubbs (27) point out, categorization results may lead a human annotator to re-think her annotation model. In this case, our error analysis seems to support the redesigned model (i.e. with **Overlap** and **Simultaneous** combined).

**Before confused as overlap.** This is the second most frequent source of confusion, accounting for 21.5% of the errors. The following example illustrates this confusion:
  She [called 911] and he was [brought] to Hahnemann General Hospital Lydia.

In this sentence, OCCURRENCE event ‘called 911’ is temporally **Before** OCCURRENCE event ‘brought’, but the relation is misclassified as **Overlap**. This source of confusion arises from the presence of the coordinating conjunction ‘and’, which frequently appears together with the **Overlap** relation. In this example, understanding that called 911 took place before bought requires world knowledge, which might be acquired via narrative chains (28).

**After confused as overlap.** This is the third most frequent source of confusion, accounting for 19.5% of the errors. The following example illustrates this confusion:
  Also, a repeat outpatient [CT colonoscopy] with [better preparation] should be considered.

In this sentence, TEST event ‘CT colonoscopy’ is proposed **After** OCCURRENCE event ‘better preparation’ in the gold standard, but the relation is misclassified as **Overlap**. The difficulty in correctly classifying this relation as **After** arises from the fact that an OCCURRENCE event can be anything that is clinically relevant to the patient’s timeline apart from the other defined attributes, and hence it can take on various temporal roles depending on whether it is in an adverbial phrase, an adjectival phrase, a noun phrase or a VP.

Intuitively, when an event happens ‘with’ another event, they generally tend to have temporal synchronicity, and in such cases entity attribute information may not be so important. However, if there isn't temporal synchronicity (as in the above sentence), then we will need to rely on information reflected by entity attributes, especially type. More specifically, to classify the relation type correctly, we will need to narrow the scope of OCCURRENCE events by including more event types that are clinically relevant to the current set of event types. These event types might include CONDITION instead of OCCURRENCE for phrases such as ‘doing well’, ‘improving’, etc., or PREP for events that are not TREATMENTS but are necessary as a step before the TREATMENT, as in the above sentence.

### Relation identification errors

For relation identification, we will perform a qualitative analysis, since it is harder to perform the kind of quantitative analysis that we did for relation classification.

One common source of errors involves cases whose relation type may be difficult even for humans to determine. Consider, for example, events listed as a sequence, as shown in the sentence below:

*[CXR], [LP], [UA] and [abdominal CT] showed no sign of infection.*

Here, the sequence of TESTS paired consecutively as CXR and LP, LP and UA, and UA and abdominal CT are unannotated in the dataset with a relation, but the identification classifier classifies them as having a relation.

Note that for sentences like this where the patient’s past history of problems is listed, it can sometimes be difficult even for a human to determine the exact temporal relation type between the events, as a mixture of temporal relations such as **Overlap**, **Before**, **After**, etc. can exist. When a case appears temporally undeterministic to a human annotator, she may choose to leave them unannotated. In other words, even though these cases are counted as errors made by our identification system, they probably shouldn’t be.

Another common source of errors involves coreferent events that appear in different sentences. Recall that we naively posit two events that have the same head as coreferent, and train a classifier to determine whether there is a relation between two such events. We noticed that this classifier classifies all instances as having a relation. However, there are many same-head events that do not have a temporal relation. To address this problem, we will need to employ a coreference classifier to determine whether two same-head events are coreferent.

The third common source of errors stems from the fact that not all temporal relations in the dataset are annotated. Consider the two sentences below:

*ESRD on HD—Pt has [ESRD] secondary to [her DM]  and is on HD.*

Pt is now [transferred] to the FICU for [further care].

In the first sentence, the PROBLEM event pair *ESRD* and *her DM*, which should have relation type **Overlap_After**, are not annotated as having a relation in the dataset, but our identification classifier determines that it does. In the second sentence, the OCCURRENCE event ‘transferred’ and the TREATMENT event ‘further care’, which should have relation type **Before**, is not annotated as having a relation in the dataset, but our identification classifier determines that they do. As in the first type of errors discussed above, even though these cases are counted as errors made by our identification system, they probably shouldn’t be.

Overall, this qualitative analysis reveals that the error rate of our relation identification system is to some extent inflated owing to the incompleteness of the gold standard annotations. Although the performance of our relation classification system significantly degrades when gold standard temporal relations are replaced by their automatically identified counterparts, we speculate that the degradation will not be as abrupt as what we currently see given a better-prepared set of gold standard annotations.

## Conclusions

We have investigated a knowledge-rich, hybrid approach to the 12-class temporal relation classification task for the clinical domain. Results on the i2b2 corpus show that when evaluated on gold standard and automatically identified temporal relations, our approach achieves a relative error reduction of 17–24% and 8–14%, respectively, over a state-of-the-art learning-based baseline.

## Appendix A

Below we show 10 of our hand-crafted rules. To understand how to interpret these rules, let us take Rule1 as an example. Rule1 says that if TREATMENT event1 and PROBLEM event2 have a semantic medical relation **TrAP**, there is a prepositional-for dependency from event1 to event2, and where both events are in numbered argument 2 related to predicate *started*.

**Table:**

Rule1	if sameSentence=TRUE &&
	event1.class=TREATMENT &&
	event1.modality=FACTUAL &&
	event2.class=PROBLEM &&
	event2.modality=FACTUAL &&
	semantic_relation(event1, event2)=**TrAP** &&
	dependency_prep_for(event1, event2) &&
	predicate(event1, Arg2, event2, Arg2)=started
	then infer relation=**Overlap**;
Rule2	if sameSentence=TRUE &&
	event1.class=PROBLEM &&
	event1.modality=FACTUAL &&
	event2.class=PROBLEM &&
	event2.modality=FACTUAL &&
	semantic_relation(event1, event2)=**PIP** &&
	predicate(event1, Arg1, event1, ArgModifer_Manner)=having
	then infer relation=**Simultaneous**;
Rule3	if sameSentence=TRUE &&
	event1.class=TEST &&
	event1.modality=PROPOSED &&
	event2.class=PROBLEM &&
	event2.modality=FACTUAL &&
	semantic_relation(event1, event2) = **PIP** &&
	event2.precedePhraseAtFiveTokenDistance = “to evaluate” &&
	then infer relation=**Overlap_After**;
Rule4	if sameSentence=TRUE &&
	event1.class=DATE &&
	event2.class=PROBLEM &&
	predicate(event1, ArgModifier_Temporal, event2, ArgModifer_Manner) = developed &&
	then infer relation=**Begins**;
Rule5	if sameSentence=TRUE &&
	event1.class=TEST &&
	event2.class=CLINCAL_DEPT &&
	event1.precedeWordAtTwoTokenDistance = “in” &&
	inPredicateArgumentRelation(event1, Arg1, event2, ArgModifer_Location) = TRUE &&
	then infer relation=**During**;
Rule6	if sameSentence=TRUE &&
	event1.class=CLINCAL\_DEPT &&
	event1.modality=FACTUAL &&
	event2.class=TREATMENT &&
	event2.modality=FACTUAL &&
	event1.precedeWordAtTwoTokenDistance = “in” &&
	inPredicateArgumentRelation(event1, Arg1, event2, ArgModifer_Location) = TRUE &&
	discourseExplicitRelationArg1Arg2(event1, event2) = Conjunction &&
	then infer relation=**During_Inv**;
Rule7	if consecutiveSentence = TRUE &&
	event1.class=OCCURRENCE &&
	event1.modality=FACTUAL &&
	event2.class=PROBLEM &&
	event2.modality=FACTUAL &&
	event1.precedeWordAtTwoTokenDistance = “after” &&
	inExplicitDiscourseRelation(event1, Arg1, event2, Arg2) = TRUE &&
	then infer relation=**Before**;
Rule8	if sameSentence=TRUE &&
	event1.class=PROBLEM &&
	event1.modality=POSSIBLE &&
	event2.class=TEST &&
	event2.modality=FACTUAL &&
	event1.followWordAtTwoTokenDistance = “on” &&
	event2.precedeWordAtTwoTokenDistance = “on” &&
	then infer relation = **Before_Overlap**;
Rule9	if sameSentence=TRUE &&
	event1.class=TREATMENT &&
	event2.class=PROBLEM &&
	event2.precedeWordAtFiveTokenDistance = “if” &&
	discourseExplicitRelationArg1Arg2(event1, event2) = Condition &&
	then infer relation = **After**;
Rule10	if sameSentence=TRUE &&
	event1.class=TREATMENT &&
	event2.class=PROBLEM &&
	predicate(event1, Arg1, event2, Arg1) = performed &&
	predicate(event1, Arg2, event2, Arg2) = underwent &&
	dependency_prep_of(event1, event2) &&
	then infer relation = **Ends**;

## Notes

1. See https://www.i2b2.org/ for more information.

2. Downloadable from http://www.hlt.utdallas.edu/∼jld082000/temporal-relations.

3. http://nlp.stanford.edu/software/corenlp.shtml.

4. To reduce the number of parameter tuning experiments, we find the *C* value that works best with the baseline classifiers and use it to train all the remaining relation classifiers in our experiments.

5. http://www.merriam-webster.com.

6. We downsample the instances belonging to the three ‘no relation’ classes by ensuring that (i) the ratio of the number of TREATMENT–PROBLEM instances to the number of ‘no relation’ instances is 0.06; (ii) the ratio of the number of TEST–PROBLEM instances to the number of ‘no relation’ instances is 0.03 and (iii) the ratio of the number of PROBLEM–PROBLEM instances to the number of ‘no relation’ instances is 0.5. These ratios are selected using held-out development data composed of 30 randomly chosen training documents.

7. For each classifier, following the order of the relations listed in Table 4, the optimal positive-to-negative instance ratios are 0.2, 0.2, 0.06, 0.2, 0.5, 1, 1, 0.3, 0.06, 0.06 and 0.09, respectively.

8. Recall that PROBLEM events have a second attribute, assertion. The value PRESENT in the node above ‘desaturate’ is its assertion value.

9. Since the dataset available to the general research community which we are using contains a subset of the documents from the dataset that was available to the shared task participants (871 discharge summaries pre-partitioned as 394 training documents and 477 test documents), we were unable to compare our system performance with any other.

10. The classifier that is being used for classifying a test instance depends on the test instance. For example, if the test instance is formed from two events that appear in the same sentence in the corresponding text, the intra-sentence event–event classifier will be used.

11. Note that under the first setting, micro *F* is equivalent to accuracy (the percentage of correctly classified test instances), since gold standard relations are used.

12. All the statistical significance tests in this article are conducted using the paired *t*-test (*P* < 0.05).

13. A closer examination of the results reveals why the lexical relations extracted from WordNet and Webster are not useful. We observed that the set of verbs used to refer to events in the discharge reports (e.g. ‘present’, ‘admit’, ‘discharge’ and ‘complain’) is fairly limited. This has made it comparatively easier to learn the temporal relations between the events they represent directly from the training data (e.g. a patient has to be ‘admitted’ first before being ‘discharged’), rendering the WordNet and Webster relations less useful.

14. For the complete list of pruning heuristics, see http://www.hlt.utdallas.edu/∼jld082000/temporal-relations.

15. If the two entities involved appear in different sentences, we create a parse tree by connecting the roots of the two parse trees in which the two entities appear to a pseudo root node.

16. In preliminary experiments on the development data, the second option yields marginally better results than the others. So the results we report in the next section are based on identification classifiers trained using the second option.

17. We attempted thresholds from *−**1.0* to *1.0* in steps of 0.1.
